# Multifaceted mitochondria in innate immunity

**DOI:** 10.1038/s44324-024-00008-3

**Published:** 2024-05-27

**Authors:** Eloïse Marques, Robbin Kramer, Dylan G. Ryan

**Affiliations:** MRC Mitochondrial Biology Unit, University of Cambridge, Cambridge, UK

**Keywords:** Mitochondria, Energy metabolism

## Abstract

The ability of mitochondria to transform the energy we obtain from food into cell phosphorylation potential has long been appreciated. However, recent decades have seen an evolution in our understanding of mitochondria, highlighting their significance as key signal-transducing organelles with essential roles in immunity that extend beyond their bioenergetic function. Importantly, mitochondria retain bacterial motifs as a remnant of their endosymbiotic origin that are recognised by innate immune cells to trigger inflammation and participate in anti-microbial defence. This review aims to explore how mitochondrial physiology, spanning from oxidative phosphorylation (OxPhos) to signalling of mitochondrial nucleic acids, metabolites, and lipids, influences the effector functions of phagocytes. These myriad effector functions include macrophage polarisation, efferocytosis, anti-bactericidal activity, antigen presentation, immune signalling, and cytokine regulation. Strict regulation of these processes is critical for organismal homeostasis that when disrupted may cause injury or contribute to disease. Thus, the expanding body of literature, which continues to highlight the central role of mitochondria in the innate immune system, may provide insights for the development of the next generation of therapies for inflammatory diseases.

## Introduction

Mitochondria are double-membraned organelles found in the cytoplasm of virtually all eukaryotic organisms. They contain their own genetic material, a circular chromosome termed mitochondrial DNA (mtDNA)^1^, differentiating them from most other eukaryotic subcellular structures with the exception of chloroplasts. It is proposed that mitochondria originated from an endosymbiotic event between an α-proteobacterial ancestor and an archaeal host of the Lokiarchaeota phylum over 2.5 billion years ago^2^, which acted as a primary driving force in eukaryotic evolution^3^. These dynamic and morphologically diverse organelles have captivated scientists for decades, inspiring several conceptual and theoretical advances across scientific disciplines, from evolution to metabolism and medicine^4^. Perhaps the most pervasive analogy for mitochondria is as the ‘powerhouse of the cell’, an analogy derived from the chemiosmotic theory of oxidative phosphorylation (OxPhos) introduced by the paradigm-shifting work of Peter Mitchell and Jennifer Moyle in the 1960s^5^. Structurally, mitochondria possess an outer mitochondrial membrane (OMM) that encloses the organelle and an inner mitochondrial membrane (IMM) that forms numerous folds called cristae, which increase the surface area available for ATP synthesis by chemiosmotic coupling. In fact, mitochondrial bioenergetics, the ability of energy-transducing pathways in mitochondria to maintain cell phosphorylation potential, is a leading theory of how endosymbiosis triggered the explosion, diversification, and multi-cellularity associated with the eukaryotic domain of life^3,6^. Equally fascinating is the idea that a break in mitochondrial endosymbiosis may even be a basis for inflammatory diseases in the modern age^7^.

The use of the terms mitochondrial function and dysfunction in the scientific literature often directly relates to mitochondrial OxPhos^8^. Although it has been proposed that this terminology is misleading and should be avoided^8^, it is also argued that these terms represent appropriate umbrella terms to describe overall mitochondrial health^9^. Despite these debates on terminology, it is clear that the powerhouse analogy only tells one part of a larger story. In the modern era, mitochondria are now known to act as central organising hubs coordinating biosynthetic and signalling modalities with the ability to influence fate and function decision-making across cell and tissue types^4,10^. This inherent complexity in mitochondrial biology has led to the proposition of mitochondria as processors of the cell and it has been suggested we refer to it as the mitochondrial information processing system (MIPs)^4^. While only time will tell if this newly suggested terminology persists, mitochondrial signal transduction is emerging as a critically important regulator of cellular and systemic physiology. This concept is perfectly illustrated in cells of our innate immune system, a universal and evolutionarily ancient form of host defence against infection and tissue damage^11^. Key components of the innate immune system include physical barriers like the skin and mucous membranes, as well as cellular and chemical defences such as phagocytes (e.g., dendritic cells (DCs), neutrophils, and macrophages), natural killer cells, and antimicrobial proteins like complement and interferons. These components work together to recognise and eliminate pathogens, initiate inflammation to recruit immune cells, and activate the adaptive immune response if needed. Our objective in this review article is to underscore the importance of mitochondrial signal transduction during the innate immune response using clear examples and a focus on phagocytes, rather than providing an exhaustive list of all studies and signals in this growing field. The integrated nature of mitochondrial physiology for the generation of these important signals will also be highlighted.

## Mitochondrial bioenergetics

### OxPhos

The electron transport chain (ETC) is a crucial component of aerobic respiration, occurring within the IMM of eukaryotic cells. The ETC consists of a series of protein complexes (I, II, III, IV) and electron carriers, including flavoproteins, cytochromes, and ubiquinone. These complexes work together to transfer electrons derived from the oxidation of redox equivalents, NADH and FADH_2_, down a series of reactions, ultimately to molecular oxygen (O_2_), the terminal electron acceptor. As electrons move along the ETC, they release energy that is utilised to pump protons (H^+^) across the IMM, establishing an electrochemical gradient known as the proton motive force (Δp). The electrochemical gradient then drives the synthesis of ATP via F_0_F_1_-ATP synthase (also known as Complex V) in a process referred to as chemiosmosis^5^. This method of maintaining cell phosphorylation potential is far superior to the other major alternative energy-transducing metabolic pathway, glycolysis^12^. The oxidation of glucose to pyruvate yields a net gain of 2 molecules of ATP per molecule of glucose, whereas the complete oxidation of glucose by OxPhos yields ~32 molecules of ATP^12^. If pyruvate is reduced to lactate in the presence of O_2_, this is commonly referred to as the Warburg effect or aerobic glycolysis, first observed in carcinoma cells^13,14^. However, it is now apparent that modulation of both OxPhos and aerobic glycolysis is a critical feature of metabolic remodelling in stimulated innate immune cells, such as macrophages and DCs.

Macrophages are phenotypically plastic phagocytic cells widely distributed throughout the body and can adopt a variety of polarisation states depending on their environment^15^. DCs on the other hand are primarily found in tissues that interface with the external environment, such as the skin, respiratory tract, and gastrointestinal tract^16^. Here, they act as important sentinels for the capture and processing of antigens to initiate adaptive immune responses. Classically activated macrophages, defined experimentally by stimulation with lipopolysaccharide (LPS) with or without interferon-gamma (IFN-γ) but can also include other microbial products, are inflammatory in nature and required to counteract pathogenic microorganisms^15^. On the other hand, anti-inflammatory macrophages, often generated experimentally using IL-4, IL-13, or IL-10 stimulation, are associated with the resolution of inflammation, wound healing, and type II immune responses^15^.

**Fig. 1:**
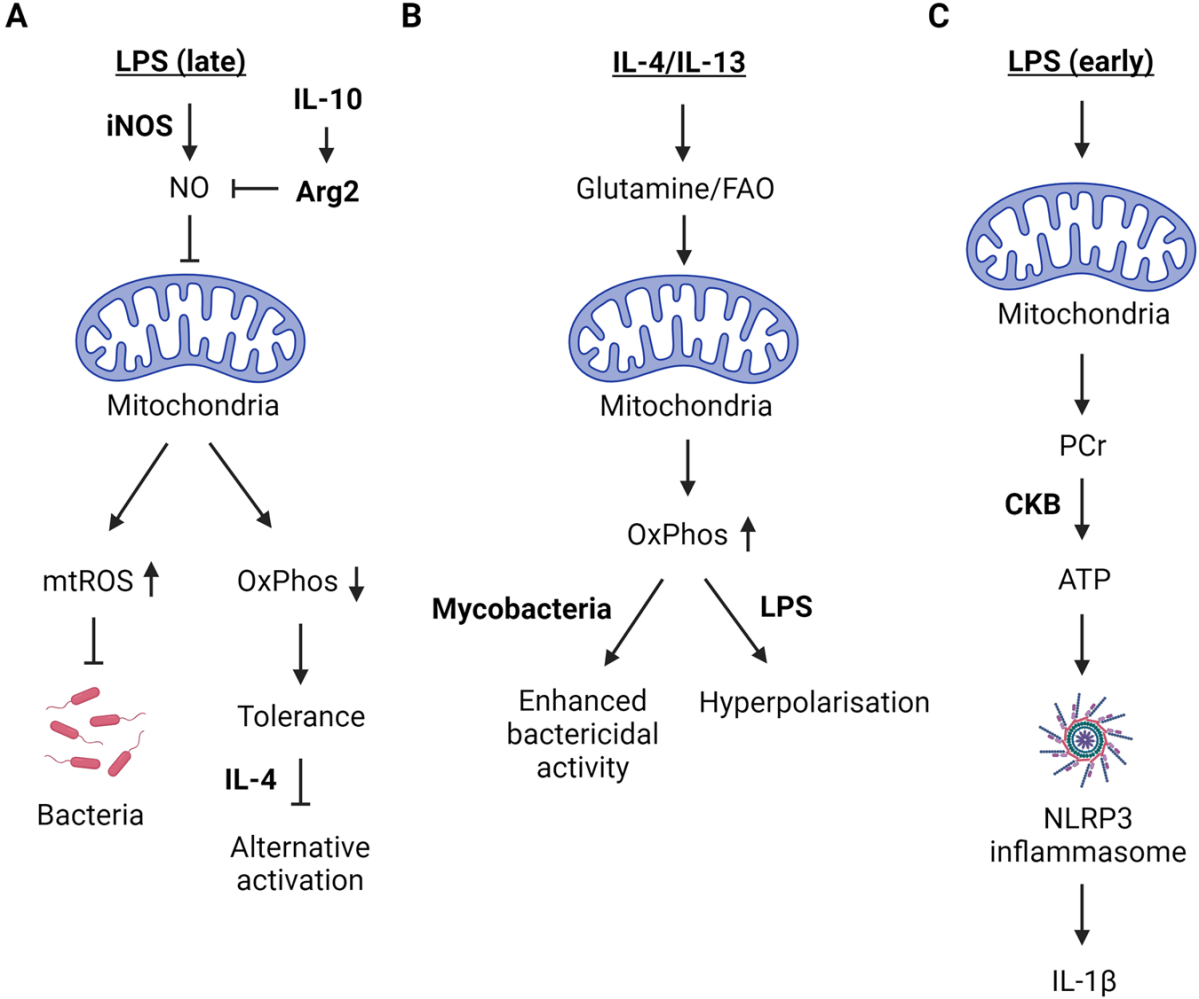
Mitochondrial bioenergetics in innate immunity. **A** NO produced by inducible nitric oxide synthase (iNOS) inhibits OxPhos and increases mtROS following prolonged LPS stimulation. Increased mtROS has bactericidal activity. Impaired OxPhos promotes tolerance and prevents alternative activation with subsequent IL-4 challenge. IL-10 antagonises NO by increasing mitochondrial Arg2. **B** IL-4/IL-13 increases OxPhos by enhancing glutamine anaplerosis and FAO. Increased OxPhos following IL-4/IL-13 training facilitates a hyperinflammatory response with subsequent LPS challenge and exhibits improved anti-mycobacterial responses. **C** NLRP3 inflammasome activation depends on mitochondrial PCr that is converted to ATP in the cytosol by CKB.

Classical activation of macrophages and stimulation of DCs by Toll-like receptor (TLR) ligands (also known as pathogen-associated molecular patterns (PAMPs)), results in the suppression of mitochondrial respiration and an increase in aerobic glycolysis (Fig. 1A)^17–20^. Mechanistically, respiratory impairment has been linked to the inducible nitric oxide synthase (iNOS), also known as NOS2, and increased nitric oxide (NO) production^18,20^. NO is a free radical that has long been known to inhibit ETC complexes in macrophages^21–24^. More recently, NO has been shown to reduce the protein levels of complexes I, II, III, and IV and impair the activity of complexes I, II, and IV in macrophages co-stimulated with LPS and IFN-γ^25,26^. In contrast, IL-4-stimulated macrophages exhibit increased OxPhos, a process dependent on PPARγ-coactivator-1β (PGC1β)-mediated mitochondrial biogenesis, CD36-dependent lysosomal lipolysis, and fatty acid oxidation (FAO) (Fig. 1B)^27,28^. As such, FAO-driven mitochondrial respiration is required for effective type II immune responses against parasitic helminth infections^28^. Intriguingly, IL-4-stimulated macrophages readily repolarise into classical inflammatory macrophages^20^. However, NO-mediated inhibition of OxPhos prevents the repolarisation of inflammatory macrophages highlighting the importance of mitochondrial bioenergetics for macrophage plasticity^20^. Similarly, the anti-inflammatory cytokine IL-10 antagonises classical macrophage polarisation by suppressing aerobic glycolysis and increasing OxPhos^29^. This positive impact of IL-10 on mitochondrial respiratory function is linked to the restriction of iNOS expression, increased arginase 2 levels, reduced NO production, and suppression of mammalian targets of rapamycin (mTOR)^29,30^.

In inflammatory macrophages or DCs, the precise reason behind the shift away from mitochondrial respiration and toward aerobic glycolysis is still unclear. This acute inflammatory response is generally short-lived when compared to the more prolonged activities of alternatively activated macrophages. Indeed, evidence suggests that inhibiting glycolysis limits the activation and survival of DCs and impairs cytokine production in macrophages, particularly the pro-inflammatory cytokine IL-1β^18,19,31^. Specifically, the rapid glycolytic burst downstream of TLR signalling in DCs facilitates anabolic processes, such as de novo fatty acid synthesis, enabling the expansion of membranes for protein secretion^32^. This suggests that the observed metabolic switch is essential for function, perhaps by allowing for the rapid synthesis of cytoplasmic ATP and reducing equivalents for these energetic processes. However, one alternative hypothesis is that by suppressing mitochondrial respiration this acts as an “off switch” for an acute inflammatory response, thereby promoting tolerance. This notion is supported by kinetic analyses of metabolic reprogramming and cytokine levels in classically activated macrophages with links to the mitohormetic impact of mitochondrial-derived reactive oxygen species (mtROS) and reactive electrophilic species (mtRES)^33,34^. In line with this concept, Garaude et al.^35^ demonstrated a transient decrease in complex I-containing super complexes and a switch to complex II-mediated mitochondrial respiration early after *E. coli* infection, which was required for anti-bacterial immunity^35^. However, at later timepoints, complex II activity had decreased relative to the uninfected controls^35^. Additional support comes from IL-4/IL-13 training of macrophages, which enhances anti-mycobacterial killing and pro-inflammatory cytokine production in a manner dependent on OxPhos^36^. Nevertheless, it can also lead to a hyperinflammatory response following subsequent LPS exposure that can potentially drive pathology^37^. Therefore, this hyperinflammatory phenotype in the absence of respiratory chain suppression may provide insights into the role of this metabolic remodelling process.

More recently, the mitochondrial ETC has also been shown to be essential for activation of the NOD-, LRR- and pyrin domain-containing protein 3 (NLRP3) inflammasome in macrophages^38^. NLRP3 serves as an intracellular sensor capable of detecting a wide array of microbial motifs, endogenous danger signals, and environmental irritants, leading to the formation and activation of the inflammasome complex^39^. This complex comprises a sensor component (NLRP3), an adaptor (ASC, also known as PYCARD), and an effector (caspase 1)^39^. Structurally, NLRP3 is a tripartite protein containing an amino-terminal pyrin domain (PYD), a central NACHT domain, and a carboxy-terminal leucine-rich repeat domain (LRR domain), with the NACHT domain exhibiting ATPase activity crucial for NLRP3 self-association and function^39^. Upon activation, the effector caspase 1 cleaves pro-IL-1β, pro-IL-18 and gasdermin D (GSDMD) to their mature forms, triggering pyroptosis and pro-inflammatory cytokine release^39^. While the mechanism by which NLRP3 senses such diverse stimuli has been extensively investigated and a direct link with mitochondria has long been established, the precise signalling involved remains unclear^40^. Billingham and colleagues utilised pharmacological inhibitors targeting complex I, II, III, and V to investigate this link and confirmed impairments in NLRP3 inflammasome activation upon inhibition of OxPhos^38^. This effect was reversed following functional complementation of complex I and complex III utilising ectopic expression of *Saccharomyces cerevisiae* NADH dehydrogenase (NDI1) or *Ciona intestinalis* alternative oxidase (AOX)^38^. Mechanistically, the authors found that mitochondrial ATP synthesis via phosphocreatine (PCr) and cytosolic creatine kinase B (CKB), which generates cytosolic ATP from PCr, was necessary for NLRP3 activation (Fig. 1C)^38^. These findings underscore the intricate interplay between OxPhos and innate immune responses.

While this study firmly connects mitochondrial bioenergetics to NLRP3, it’s important to note some conflicting reports. NLRP3 inflammasome activation can occur in a K^+^ efflux-dependent and K^+^ efflux-independent manner^41,42^. K^+^ efflux-dependent activation is reportedly unrelated to mitochondrial bioenergetics^41^, while Imiquimod and CL097 trigger K^+^ efflux-independent NLRP3 activation by inhibiting mitochondrial complex I^41^. However, complex I inhibition or PCr depletion prior to CL097 treatment still impaired IL-1β release, suggesting additional mechanisms are involved during CL097 signalling^38^. Furthermore, treatment of macrophages with the complex II inhibitor malonate increased intracellular succinate levels but had a modest impact on IL-1β release^43^. However, malonate is a negatively charged dicarboxylate with poor membrane permeability^44^. It is unclear how malonate enters the cell at neutral pH, what intracellular concentrations of malonate were achieved, or if it led to a significant impairment in mitochondrial respiration. As such, the use of multiple ETC inhibitors alongside measures of respiration by Billingham et al.^38^ provides solid evidence for the involvement of mitochondrial OxPhos. Despite this, important questions remain regarding why ATP produced by glycolysis or direct ATP export to the cytosol via the adenine nucleotide transporter (ANT) are insufficient to support NLRP3 activity. The data also suggests that there is no role for mtROS in NLRP3 activation in contrast to previous reports^40,45–49^. This highlights the importance of conducting further research into this complex process in order to clarify underlying mechanisms and to aid any potential therapeutic targeting in the future.

### mtROS

ROS are chemically reactive molecules containing oxygen, traditionally thought of as agents of cellular damage. Indeed, cytosolic ROS produced by NADPH oxidase 2 (NOX2) in innate immune cells are known to directly damage pathogens through the oxidation of lipids and DNA^50–53^. Beyond these NOX enzymes, which evolved as anti-microbicidal tools of phagocytes^54^, a consequence of the use of mitochondrial OxPhos for energy transduction is the generation of mtROS^55,56^. mtROS, notably superoxide (O_2_^•−^) and, following dismutation, hydrogen peroxide (H_2_O_2_), are predominantly formed at complex I or complex III of the ETC^55^. The significant contribution of mtROS to inflammatory redox signalling in innate immune cells, as well as anti-microbial immunity, has become increasingly prominent over the years^57–59^.

For instance, stimulation of Toll-like receptors (TLR) 1,2, and 4 on the surface of innate immune cells and within their phagosome initiates various signalling pathways within the cell^60^. Among others, it causes mitochondrial migration towards the phagolysosome through the activation of the serine-threonine kinases Mst1 and Mst2^57,61–63^. Simultaneously, West et al.^57^ observed mtROS production in macrophages following cell-surface TLR stimulation (Fig. 1)^57^. Interestingly, the production of mtROS that was induced by TLR binding is specific to antimicrobial defence, as it was not observed after stimulation of endosomal TLRs that function primarily in antiviral defence^57^. In response to a methicillin-resistant *Staphylococcus aureus* infection, mtROS production is also stimulated, leading to the formation of mitochondria-derived vesicles (MDVs)^64^. These MDVs delivered mitochondrial matrix enzyme manganese superoxide dismutase (SOD2) to bacteria-filled phagosomes, enhancing bacterial clearance.

Beyond their direct antimicrobial effects, infection-induced mtROS can trigger the production of pro-inflammatory cytokines. Herb et al.^65^ demonstrated that *Listeria monocytogenes* infected murine macrophages generate mtROS, which enter the cytosol and induce secretion of pro-inflammatory cytokines^65^. Likewise, complex I-derived mtROS are implicated in the stabilisation of hypoxia-inducible factor 1 alpha (HIF-1α) and expression of IL-1β downstream of prolonged TLR4 activation^59,66^. As previously mentioned, mtROS have been repeatedly implicated in the activation of the NLRP3 inflammasome and the subsequent maturation of IL-1β and IL-18, through an indirect mechanism that will be discussed further on^40,45,46,48,49,67,68^. Interestingly, a gain-of-function mutation in leucine-rich repeat kinase 2 (Lrrk2^G2019S^), which is associated with familial Parkinson’s disease, leads to increased mtROS and a functional switching of cell death pathways in macrophages^69^. Specifically, mtROS redirects GSDMD to mitochondrial membranes triggering a switch to necroptosis and a hyperinflammatory response to *Mycobacterium tuberculosis* infection^69^. In agreement, ROS-mediated oxidation of cysteine 192 in GSDMD has also been shown to promote GSDMD oligomerisation and pyroptotic cell death^70^. The idea that mtROS are pro-inflammatory in nature is further supported by the anti-inflammatory action of mitophagy. Mitophagy serves as a protective mechanism against excessive mtROS by selectively degrading damaged mitochondria, as observed with IL-10 antagonism of LPS triggered inflammation^30^, while the absence of autophagy also results in ROS-dependent amplification of retinoic acid-inducible gene I (RIG-I)-like signalling^71–73^.

Despite the emerging importance of mtROS signalling, the relative contribution of complex I versus complex III to mtROS generation in inflammatory macrophages is currently a topic of debate^56,59,74^. The role of complex I in OxPhos is to harvest electrons from NADH and transfer them to the ubiquinone (CoQ) pool while pumping protons across the IMM. This forward electron transfer (FET) will occur if the difference in reduction potential between the NAD^+^/NADH and the CoQ/CoQH2 couples (ΔE_h_) is sufficient to pump protons against Δp, which is composed of the mitochondrial membrane potential (ΔΨ_m_) and pH gradient (i.e. 2ΔE_h_ > 4Δp)^56^. When 4Δp > 2ΔE_h,_ electrons can also be transferred in the reverse direction, known as reverse electron transport (RET), from the CoQ pool through complex I to flavin mononucleotide (FMN), and subsequently passed to O_2_ to generate O_2_^•−^^56^. Indeed, current evidence favours this model of activation downstream of TLR4 activation, albeit from indirect measurements^58,59,66^. On the other hand, complex III transfers electrons from CoQH2 to cytochrome c (cyt c) via the Q-cycle and can generate O_2_^•−^ at the Q_o_ site^56^. However, the physiological relevance of O_2_^•−^ production at complex I is thought to be higher than that of complex III^55,56^. Despite this, complex III-derived mtROS is reported to drive oxidative DNA damage in macrophages enforcing reliance on NAD^+^ salvage pathways to sustain aerobic glycolysis and pro-inflammatory cytokine production^74^. The evidence for complex III mtROS derives primarily from the use of Q_o_ site inhibitor myxothiazol, while showing no impact of rotenone^74^. However, since RET is dependent on 4Δp > 2ΔE_h,_ which will be impacted by complex III inhibition, the use of this compound cannot exclude mtROS production at complex I^56^.

Mitochondria in neutrophils, historically undervalued due to their preference for glycolysis, have recently gained recognition for their involvement in neutrophil extracellular traps (NETs), motility, degranulation, and respiratory burst^75^. The production of ROS by neutrophils during the respiratory burst is a key mechanism for regulating infection and inflammation^75^. While mtROS do not directly contribute to intracellular ROS stores, it is implicated in the oxidative burst caused by NOX2 activation and degranulation^76^. Notably, production of mtROS regulates neutrophil motility in vivo, as demonstrated by Zhou et al. using a zebrafish model^77^.

NETs are complex networks comprised of modified chromatin and bactericidal proteins, which were initially associated with cell death in a process termed NETosis. It is now recognised that NETosis exists in two forms: the prolonged ‘suicidal’ NETosis and the rapid ‘vital’ NETosis that leaves neutrophils alive^78–80^. Classically, NETosis was believed to be dependent on ROS produced by cytosolic NOX2^81^. However, Douda et al.^82^ and Reithofer et al.^83^ elucidated the mechanisms behind a second NOX-independent NETosis type, demonstrating that calcium (Ca^2+^)-dependent NETosis requires Ca^2+^ influx from lysosomes or the extracellular space. Mitochondria sense these elevated Ca^2+^ levels, generating mtROS. Both cytoplasmic Ca^2+^ and mtROS generated at complex III of the ETC are required for activation of peptidyl arginine deiminase 4 (PAD4), crucial for chromatin decondensation and NETosis^82–84^. However, the evidence for complex III-derived O_2_^•−^ was determined from the use of the Q_i_ site inhibitor antimycin A^56^. There was no decrease observed with myxothiazol or the complex III-specific O_2_^•−^ suppressor, S3QEL^84,85^. As such, the source of mtROS in neutrophils remains to be definitively determined. Finally, NETs containing mtDNA oxidised by mtROS induce high levels of type I interferon (IFN) signalling and are reported to contribute to systemic lupus erythematosus (SLE)^86,87^.

This emerging role for mtROS in inflammation and anti-microbial activity highlights a critical repurposing of mitochondrial function away from OxPhos toward redox signalling. However, many open questions remain about how such signals propagate from mitochondria in the presence of abundant anti-oxidants to engage their reported targets in different cellular compartments. One hypothesis posits that mtROS signalling to the cytosol is achieved by localised redox relays involving peroxiredoxins and glutathione peroxidases^88,89^, which remains to be explored in the context of innate immune signalling. Alternatively, a second proposal is the floodgate model, which involves the inactivation of scavenging enzymes, enabling the oxidation of target proteins by H_2_O_2_^89^. While redox signalling may be important for cellular and organismal homeostasis, it can also contribute to disease pathology under certain circumstances^59,90^ and so identifying the source of mtROS will be a critical question to address in the future. To elucidate the source of mtROS in innate immune cells, genetic models will likely be required. One model, the ND6 G14600A mtDNA mutation, which leads to a proline to leucine substitution at position 25 in the ND6 subunit of complex I (ND6-P25L), may be used in the future^56,91^. Importantly, the mutant complex I is fully active for NADH oxidation and has little impact on FET, but cannot generate ROS by RET^91^. It also protects the heart from ischaemia-reperfusion (I/R) injury, a process driven by succinate oxidation and O_2_^•−^ production by RET^90–92^.

### Mitochondrial membrane potential (ΔΨ_m_) and Ca^2+^

In addition to its role in maintaining cell phosphorylation potential, ΔΨ_m_ is indispensable for multiple aspects of mitochondrial physiology, including mtROS production and the transport of many proteins, metabolites, and ions^56,93^. Mills et al. have highlighted that LPS stimulation augments ΔΨ_m_ in macrophages, which together with the enhanced oxidation of succinate by complex II, results in accumulation of mtROS and elevated *Il1b* gene expression^59^. Conversely, alternatively activated IL-4-stimulated macrophages exhibit a dissipated ΔΨ_m_ when treated with the lipid immunomodulator prostaglandin E2 (PGE2)^94^. Mechanistically, PGE2-induced dissipation of ΔΨ_m_ was related to the malate-aspartate shuttle and led to voltage-dependent changes in gene expression, partly regulated by the transcription factor ETS variant 1 (ETV1)^94^. These studies are noteworthy as they provide evidence that external stimuli, in this case LPS and PGE2, can alter ΔΨ_m_, thereby inducing mitochondria-to-nucleus retrograde communication and fine-tuning macrophage polarisation states.

Moreover, other roles for ΔΨ_m_ have recently emerged in different innate immune subsets. Efferocytosis, the successful clearance of apoptotic cells by phagocytes, effectively doubles the content of the engulfing cell, thereby introducing many more metabolites^95^. Park and colleagues illustrated that the mitochondrial membrane protein uncoupling protein 2 (UCP2), which lowers ΔΨ_m_, is essential for the functional clearance of apoptotic target cells but not for the clearance of synthetic targets^96^. Similarly, aged DCs that exhibited lower ΔΨ_m_ and coupling efficiency were less efficient at endocytosing irradiated cells and cross-presenting antigens to T cells than their younger counterparts^97^. This effect of reduced ΔΨ_m_ on antigen processing and presentation has also been observed as a result of physiological carbon monoxide production^98^. Furthermore, inducing mitochondrial dysfunction in younger DCs diminished their phagocytic and cross-presenting capacity, whereas mtROS specifically affected cross-presentation. This aligns with the work of Oberkampf and colleagues, who demonstrated that mtROS regulate cross-presentation to cytotoxic T cells by plasmacytoid DCs (pDCs)^99^.

Another important aspect of the ΔΨ_m_ is its role as the driving force behind the uptake of Ca^2+^ into the mitochondrial matrix. Cytosolic Ca^2+^ serves as a pivotal intracellular signalling messenger, implicated in processes such as exocytosis, cell motility, and apoptosis^100^. Regulation of cytosolic Ca^2+^ primarily occurs through Ca^2+^ uptake from the extracellular space and release from organelles, such as the endoplasmic reticulum. Elevated levels of cytosolic Ca^2+^ trigger Ca^2+^ influx into the mitochondrial matrix through the mitochondrial calcium uniporter complex (MCU), thereby buffering cytosolic Ca^2+^ and regulating mitochondrial respiration^93^. This complex consists of the channel-forming subunit MCU and its regulators MICU1, MICU, MCUb, EMRE, MCUR1 and miR-25^101^.

The MCU has been the subject of many studies investigating its role in macrophage function. For instance, the MCU functions as a regulator of phagocytosis-dependent NLRP3 inflammasome activation in response to bacterial challenges^102–105^. Mechanistically, mitochondrial Ca^2+^ uptake inhibits endosomal sorting complex required for transport (ESCRT)-mediated phagolysosomal membrane repair, which enables NLRP3 activation^105^. Additionally, expression of *MCU* and *MICU1* inversely correlate with age, resulting in reduced mitochondrial Ca^2+^ uptake in aging macrophages^106^. This leads to an amplification of cytosolic Ca^2+^ oscillations, a major driver of nuclear factor kappa B (NF-κB) activation and inflammation. Interestingly, the abundance of the dominant-negative subunit MCUb is associated with macrophage polarisation during skeletal muscle regeneration, indicating that the composition of the MCU complex influences macrophage phenotypes^107^. This was underscored by Lu et al.^108^, who investigated the role of the MCU in atherosclerosis-mediated efferocytosis dysfunction. Using an MCU-specific inhibitor, they were able to attenuate the upregulation of MCU and MCUR1 and the downregulation of MCUb induced by oxidised low-density lipoprotein, which coincided with reduced production of ROS and pro-inflammatory cytokine and improved efferocytosis^108^. In DCs, circadian changes in mitochondrial Ca^2+^ have also been found to regulate antigen processing and T cell activation^109^. These rhythmic changes in mitochondrial Ca^2+^ were driven by the circadian control of key regulators of the mitochondrial calcium uniporter complex, including MCUb and EMRE.

Finally, recent work by Monteith et al.^110^ demonstrated that the MCU, and the resulting Ca^2+^ flux, steers neutrophils away from primary degranulation and towards suicidal NETosis^110^. Murine neutrophils deficient in MICU1 exhibited increased bactericidal activity, particularly in the presence of macrophages or during systemic *S. aureus* infection^110^. Moreover, activation of the MCU and mitochondrial Ca^2+^ uptake promotes neutrophil polarisation and chemotaxis, further emphasizing the critical importance of mitochondrial Ca^2+^ dynamics in innate immune cells^111^. All of these studies on ΔΨ_m_ and Ca^2+^ together illustrate how virtually all key effector functions of innate immune cells are governed by mitochondrial physiology and strongly illustrate the concept of mitochondria as an information processing system.

## Mitochondrial nucleic acid signalling

### mtDNA

Mitochondrial nucleic acids encompass the entire genetic material found within mitochondria, which includes mtDNA and mitochondrial RNA (mtRNA). The primary component, mtDNA, exists in multiple copies within each mitochondrion, with the number varying depending on the cell type and energy demand^1^. In humans, mtDNA consists of a circular, double-stranded molecule containing approximately 16,500 base pairs^1^. Unlike nuclear DNA, mtDNA is only inherited matrilineally, reflecting its unique evolutionary history and mode of transmission^1^. Within mtDNA, there are 37 regions encoding essential genes critical for mitochondrial function, including 13 subunits of the ETC involved in OxPhos, as well as transfer RNAs (tRNAs) and ribosomal RNAs (rRNAs) necessary for mitochondrial translation^1^. However, this only represents a minor component of the mitochondrial proteome, with the remaining 99% encoded by the nuclear genome^112^. The similarities between eukaryotic mtDNA and bacterial DNA is a key piece of evidence for the endosymbiotic origin of mitochondria. However, these properties also allow mitochondrial signals to act as endogenous danger-associated molecular patterns (DAMPs) to drive inflammation^113–115^.

**Fig. 2:**
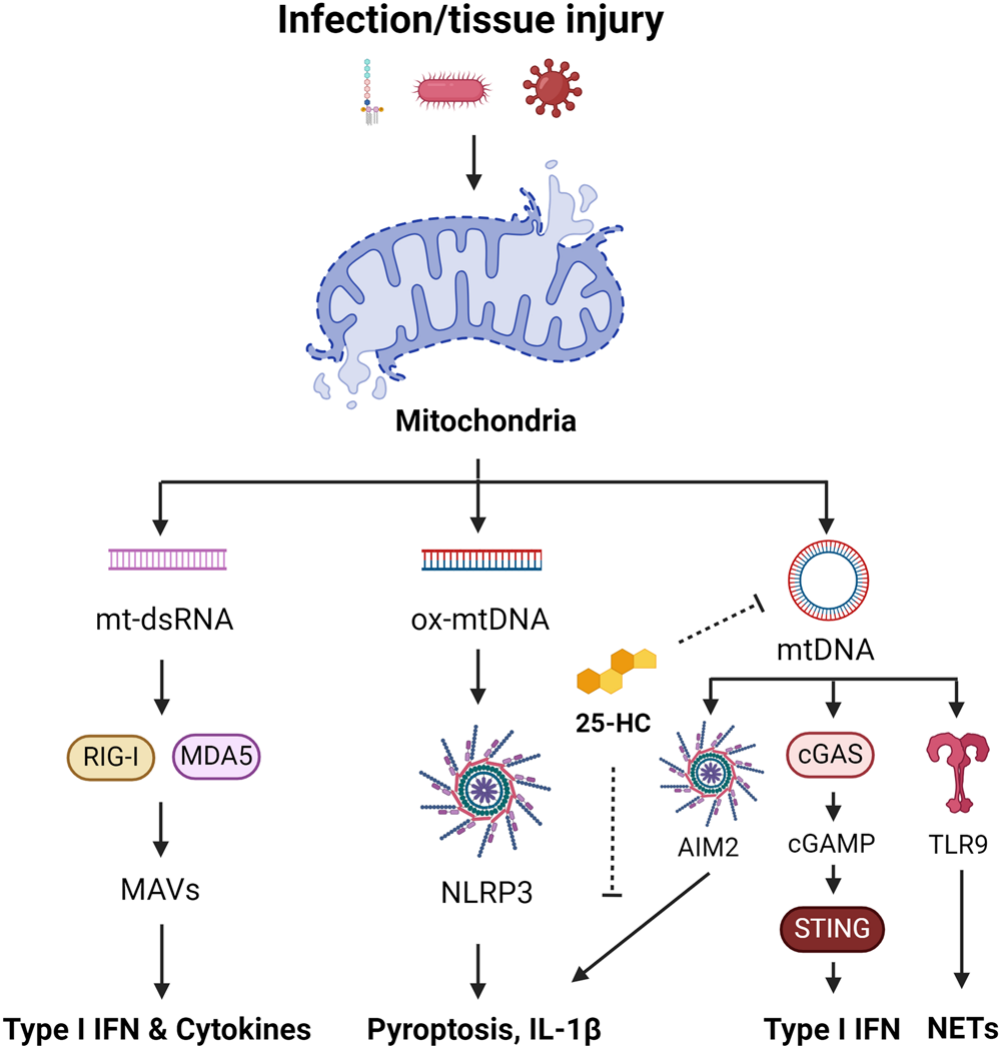
Mitochondrial nucleic acid signalling in innate immunity. Infection with bacteria or viruses, as well as tissue injury, can lead to mitochondrial damage and the release of mitochondrial nucleic acids, including mtDNA and mt-dsRNA. mt-dsRNA can be sensed by RIG-I and MDA5, which signal via MAVS, to promote the expression of type I IFN and pro-inflammatory cytokines. Ox-mtDNA is a reported ligand for the NLRP3 inflammasome triggering pyroptosis and IL-1β maturation. mtDNA also activates the AIM2 inflammasome, cGAS-STING pathway, and TLR9 to drive type I IFN and NETosis. 25-HC can inhibit mtDNA release and AIM2 activation arising from elevated cholesterol.

mtDNA, akin to bacterial DNA, possesses a significant proportion of hypomethylated CpG dinucleotides, which are motifs recognised by TLR9 to trigger an innate immune response^116^. Tissue injury resulting from trauma can induce a systemic inflammatory response syndrome (SIRS), which shares clinical similarities with sepsis^115^. In SIRS, the release of mitochondrial DAMPs, including *N*-formyl peptides and mtDNA, activate polymorphonuclear neutrophils (PMNs)^115^. This activation leads to degranulation and organ injury following TLR9 sensing of mtDNA (Fig. 2)^115^. Additionally, mtDNA and TLR9 activation drive NET formation and lung injury during primary graft dysfunction after lung transplantation^117^. Furthermore, previous research by Oka et al.^118^ demonstrated that mtDNA escape from autophagy in cardiomyocytes contributes to TLR9-mediated inflammation and subsequent heart failure^118^. Collectively, these studies suggest that TLR9 sensing of mtDNA is essential for driving pathological sterile inflammation following injury.

In addition to TLR9, mtDNA can also be detected by other intracellular sensors such as the absence in melanoma 2 (AIM2) inflammasome^119^, the NLRP3 inflammasome^46–49^, and the cyclic GMP–AMP synthase (cGAS)-stimulator of interferon response cGAMP interactor 1 (STING) pathway (Fig. 2)^120^. AIM2, a cytosolic DNA sensor, triggers the maturation of IL-1β and pyroptosis in response to mtDNA release^119,121^. Research by Dang et al.^119^ highlights the role of 25-hydroxycholesterol (25-HC) in limiting cholesterol-dependent mtDNA release following bacterial infection or LPS stimulation in macrophages^119^. This suggests that macrophages employ mechanisms to preserve mitochondrial integrity and prevent excessive AIM2-mediated inflammation.

While AIM2 can sense mtDNA, newly synthesized and oxidised mtDNA (ox-mtDNA) fragments are reported to activate the NLRP3 inflammasome, driving the processing of IL-1β^38^. Recent in vitro studies propose that the pyrin domain of NLRP3 shares a protein fold with DNA glycosylases, potentially enabling recognition of ox-mtDNA^122^. However, an unidentified mediator may also be involved. Zhu and colleagues identified an orphan receptor, Nur77, which binds both intracellular LPS and mtDNA, leading to non-canonical NLRP3 activation^123^. This mechanism was found to be independent of canonical activation and unlikely to serve as an ox-mtDNA receptor for canonical NLRP3 signalling. Recent findings have also cast doubt on the role of mtROS production in canonical NLRP3 inflammasome activation^38^. As such, further research is required to understand the precise role of mtROS and ox-mtDNA in this signalling axis.

cGAS functions as both a nuclear and cytosolic protein, responding to cytosolic double-stranded DNA by catalysing the formation of cGAMP, a second messenger that initiates an inflammatory response via STING^120,124^. The activation of STING by cGAMP promotes type I IFN production via the transcription factor interferon-regulatory factor 3 (IRF3), initiating an antiviral immune response. The cGAS-STING pathway plays a crucial role in sensing intracellular pathogens, including *M. tuberculosis*^125^, herpesvirus^120^, dengue virus^126^, norovirus^127^, influenza A virus^128^, encephalomyocarditis virus^128^ and severe acute respiratory syndrome coronavirus 2 (SARS-CoV2)^129^, which all promote the release of mtDNA to enhance detection and antiviral signalling. Moreover, cGAS is also involved in detecting extracellular bacteria including *Pseudomonas aeruginosa, Klebsiella pneumoniae, and Staphylococcus aureus*^130^. These findings indicate that the surveillance of mitochondrial integrity cooperates with viral and bacterial sensing mechanisms to fully engage the host innate immune response. However, in the case of SARS-CoV2 and coronavirus disease (COVID19), this can lead to severe lung inflammation and pathology, primarily driven by macrophages^129^.

### mtRNA

The process of mtDNA transcription and translation occurs primarily within the mitochondria, facilitated by the mtRNA machinery. mtRNA includes precursor transcripts that undergo processing to produce mature mRNAs, tRNAs, and rRNAs, which are essential for mitochondrial protein synthesis. A consequence of the bacterial origin of circular mtDNA is that it is subject to bidirectional transcription, which generates overlapping transcripts capable of forming long double-stranded RNA (dsRNA) structures^131–133^.

Similar to mtDNA, mitochondrial dsRNA (mt-dsRNA) has been found to trigger a type I IFN response mediated by the cytosolic viral RNA sensors, RIG-I^134^ or melanoma differentiation-associated protein 5 (MDA5) (Fig. 2)^133^. Deletion of the autophagy protein IRGM1 in macrophages has been shown to impair mitophagy and drive inflammation via TLR7 signalling^135^. TLR7, an endosomal TLR, senses viral and bacterial single-stranded RNA (ssRNA)^136,137^, suggesting it may also sense mtRNA following mitochondrial damage. Supporting this notion, inhibition of the TCA cycle enzyme fumarate hydratase (FH) in LPS-stimulated macrophages, which impairs mitochondrial respiration, is reported to drive IFNβ release via the combined action of RIG-I, MDA5 and TLR7^138^. However, further work is required to determine if this is the case. Suppression of inflammatory mitochondrial RNA species also appears crucial to prevent autoimmunity. Defects in RNA editing by ADAR1^139^, essential to prevent dsRNA/MDA5-mediated inflammation, and TLR7 gain-of-function mutations^140^, are previously underappreciated mechanisms of common inflammatory diseases, such as SLE.

Together, these studies provide compelling evidence that mitochondrial nucleic acid signalling regulates host innate immune responses to resolve the infection. However, these signalling events must be tightly controlled in order to prevent immunopathology. This dual role reinforces the concept of mitochondria as sequestered processors within the cell, highlighting the importance of maintaining this endosymbiotic relationship^7^.

## Mitochondrial metabolite and lipid signalling

### TCA cycle remodelling, signalling and anti-microbial action

The tricarboxylic acid cycle (TCA cycle), also known as the citric acid cycle or the Krebs cycle, is a fundamental aspect of cellular metabolism^141^. Consisting of a series of enzymatic reactions, the TCA cycle plays a crucial role in extracting energy from carbohydrates, fats, and proteins to produce NADH and FADH_2_, which then fuel the ETC for ATP synthesis^141–143^. Besides its energy-generating function, the TCA cycle contributes to biosynthetic processes by providing precursors for the synthesis of amino acids, nucleotides, and other essential biomolecules^143^. Recent studies have also revealed intricate connections between TCA cycle remodelling and innate immunity, unveiling a novel dimension of immune regulation^19,33,142,144–146^. Importantly, metabolic intermediates generated from the TCA cycle, or TCA cycle metabolites themselves, serve as signalling molecules that modulate immune responses beyond their roles in bioenergetics or biosynthetic pathways, which have been extensively reviewed elsewhere^142,147–150^. Here, we will highlight several key findings in this area of research focusing on the mechanisms and kinetics of TCA cycle remodelling, and the anti-microbial and/or signalling roles of α-ketoglutarate, succinate, fumarate, and itaconate.

**Fig. 3:**
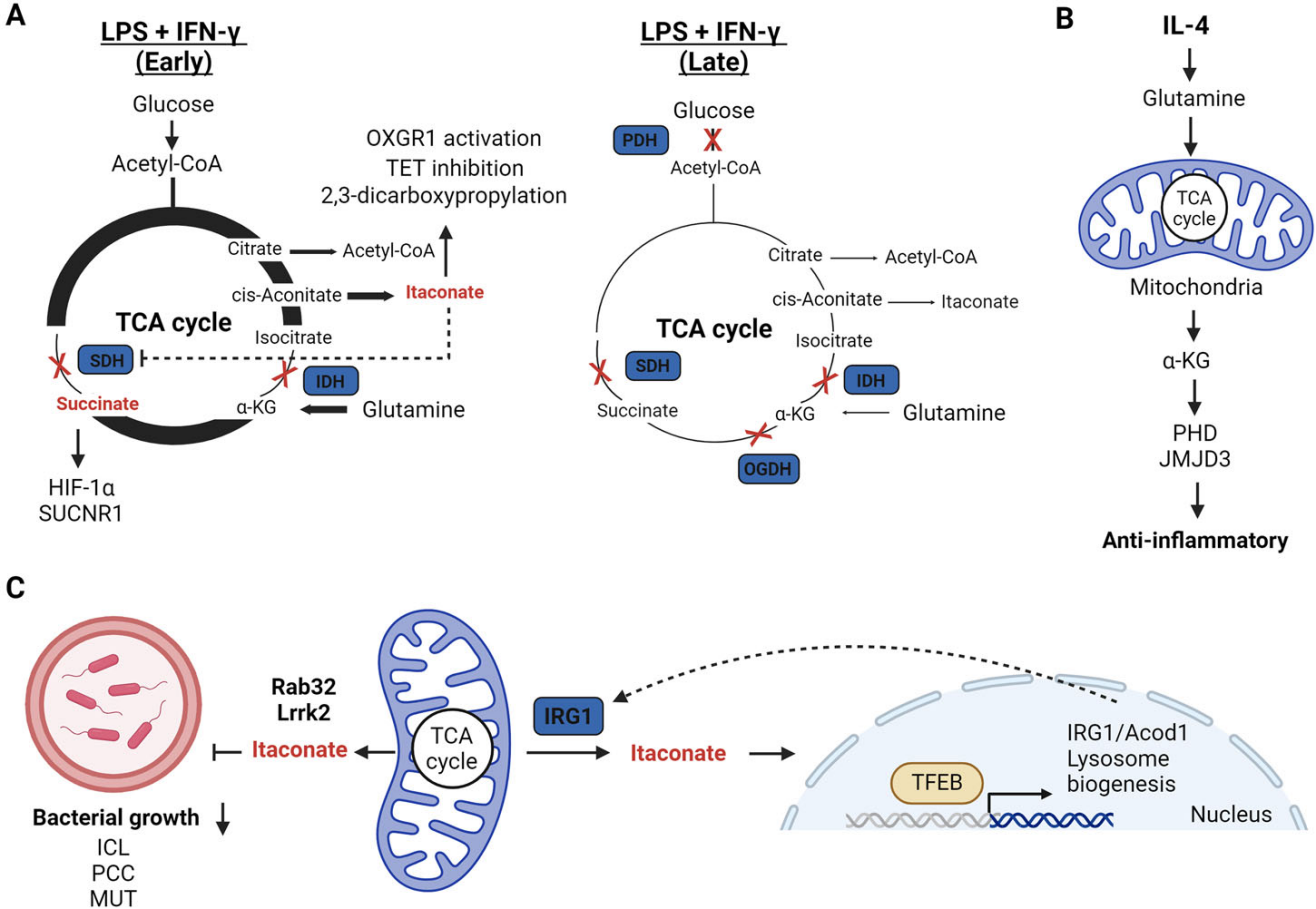
Mitochondrial metabolite signalling and anti-bacterial activity in innate immunity. **A** TCA cycle remodelling during early and later phases of stimulation post LPS and IFN-γ. Early- to mid-phase changes lead to increased itaconate and succinate levels that can signal through a variety of mechanisms before decreasing at a later stage. **B** IL-4-mediated increases in glutamine anaplerosis leads to high levels of α-KG that promotes anti-inflammatory gene expression via increased PHD and JMJD3 activity. **C** Mitochondrial-derived itaconate is trafficked into phagolysosomes in a TFEB, Rab32, Lrrk2-dependent manner where it is directly anti-bactericidal via inhibition of ICL, PCC, or MUT. Itaconate also activates TFEB to promote lysosome biogenesis.

As discussed earlier, inflammatory macrophages and DCs suppress OxPhos in a manner dependent on NO production^18,20^. However, TCA cycle remodelling downstream of TLR4 activation occurs in stages eventually leading to the initial accumulation of succinate and itaconate, followed by their decrease after prolonged stimulation (Fig. 3A)^19,138,145,151^. This process is reported to occur in two stages^33^, but a case for three stages could also be made^152^. In the first stage, LPS stimulation transiently increases mitochondrial respiration^152^. This stage is dependent on the mitochondrial glycerol 3-phosphate dehydrogenase (GPD2), a component of the glycerol phosphate shuttle, which enhances glucose oxidation to fuel acetyl-CoA-mediated histone acetylation of key inflammatory genes^152^. Importantly, acetyl-CoA is synthesized by the ATP-citrate lyase in the cytosol from mitochondrial-derived citrate^152–154^. After this first stage, two different breakpoints of the TCA cycle have been proposed. The first metabolic breakpoint occurs at isocitrate dehydrogenase (IDH), while the second break point occurs at complex II, also known as succinate dehydrogenase (SDH) (early)^33,144^. The breakpoint at IDH has been attributed to the decreased expression and activity downstream of autocrine type I IFN signalling^155^. Conversely, the breakpoint at complex II has been attributed to immunoresponsive gene 1 (IRG1), also known as *cis*-aconitate decarboxylase (CAD), mediated itaconate synthesis. Itaconate acts as a weak competitive inhibitor of complex II^145,146,151^ and is also reported to inhibit IDH2^156^, linking itaconate to both TCA cycle breakpoints. The third stage of TCA cycle reprogramming (late) is largely driven by the inhibition of pyruvate dehydrogenase complex (PDHC) and the oxoglutarate dehydrogenase complex (OGDC)^33^. Mechanistically, this is controlled by dynamic changes in the lipoylation state of both PDHC and OGDC E2 subunits and phosphorylation of the PDHC E1 subunit^33^. Additionally, this may be linked to NO production, which inhibits the TCA cycle enzyme aconitase 2 (ACO2) and PDHC^25,157^. These two stages of metabolic reprogramming are crucial mechanisms to support acute phase inflammation and restrict a hyperinflammatory response.

The accumulation and release of succinate has emerged as a crucial signal influencing innate immune responses in both normal and pathological states. In macrophages, elevated levels of succinate are reportedly exported from mitochondria to the cytosol during TCA cycle rewiring, stabilising HIF-1α and thereby promoting the synthesis of pro-inflammatory cytokines such as IL-1β^19,33^. HIF-1α stability is regulated by prolyl hydroxylases (PHDs), which target it for degradation by the proteasome, requiring α-KG as a substrate. Notably, α-KG has demonstrated the ability to dampen the activation of pro-inflammatory macrophages, supporting endotoxin tolerance post-activation (Fig. 3B)^158,159^. Mechanistically, α-KG suppresses IKKβ and NF-κB in a PHD-dependent manner and impedes the stabilisation of HIF-1α^158,159^. Thus, a reduced α-KG:succinate ratio is associated with the pro-inflammatory phenotype. In contrast, glutamine-derived α-KG is also required for alternative macrophage activation driving FAO and Jmjd3-dependent epigenetic reprogramming of IL-4 target genes^158^. Succinate oxidation at complex II within mitochondria is also proposed to drive mtROS from complex I by RET, thereby stabilising HIF-1α^92^. Given the reports of complex II inhibition by itaconate and dimethyl malonate (DMM), which increase succinate levels, reduce HIF-1α, and limit IL-1β^43,59,145^, succinate oxidation is likely a stronger driving force for HIF-1α stabilisation than succinate accumulation per se and requires further investigation. In DCs, succinate is associated with the mobilisation of intracellular Ca^2+^, leading to migratory responses and acting synergistically with TLR ligand stimulation to produce pro-inflammatory cytokines^160^. In this instance, succinate drives this response via binding to its G-protein coupled receptor succinate receptor 1 (SUCNR1), also known as GPR91, on the cell surface. Strikingly, extracellular liver succinate can drive inflammation and non-alcoholic fatty liver disease (NAFLD), which indicates succinate can act as a mitochondrial DAMP^161^. However, succinate-SUCNR1 can also promote hyperpolarisation of anti-inflammatory macrophages^162^ and decrease inflammatory markers in adipose tissue^163^, suggesting that succinate signalling is context specific.

Itaconate has emerged as a potent immunoregulatory metabolite primarily synthesized by cells of the myeloid lineage^150^. In macrophages, itaconate plays dual roles as a potent anti-bactericidal metabolite and an immunomodulator that restricts the production of pro-inflammatory cytokines (Fig. 3C)^145,150,151^^,164^. Previously, the anti-bacterial properties of itaconate were attributed to its interference with bacterial growth through the inhibition of enzymes such as isocitrate lyase (ICL) in the glyoxylate cycle^151,165^ or propionyl-CoA carboxylase (PCC) in the citramalate cycle^166^. More recently, itaconate was reported to undergo conversion into the CoA derivative itaconyl-CoA^167^, which limits *M. tuberculosis* growth by inhibiting B_12_-depencent methylmalonyl-CoA mutase (MUT)^168^. As such, itaconate can target multiple enzymes of pathogen propionate metabolism to enforce nutrient stress. To combat intracellular bacteria such as *Legionella*^169^ and *Salmonella*^170^, mitochondrial-derived itaconate is delivered to phagolysosomes. In the case of *Salmonella* infection, this host defence mechanism relies on a scaffolding complex involving mitochondria, IRG1/CAD, the GTPase Rab32, Lrrk2 and *Salmonella*-containing vacuoles (SCVs)^170,171^. This crosstalk between mitochondria and phagolysosomes is dependent on the lysosomal biogenesis factor transcription factor EB (TFEB)^172^. Itaconate, in turn, induces lysosome formation by disrupting mTOR/14-3-3-mediated cytosolic retention of TFEB^173^. Therefore, itaconate is both a direct anti-bactericidal agent and co-ordinator of cellular lysosomal signalling. However, many pathogens have evolved intricate mechanisms in an attempt to evade the anti-bacterial action of itaconate. For instance, *Yersinia pestis* and *Pseudomonas aeruginosa* encode the enzymes itaconate CoA transferase, itaconyl-CoA hydratase, and (S)-citramalyl-CoA lyase that metabolise itaconate to pyruvate and acetyl-CoA and promote their survival in macrophages^174^. Conversely, *M. tuberculosis* encode the bifunctional enzyme β-hydroxyacyl-CoA lyase required for itaconate and leucine catabolism^175^. This nicely highlights the evolutionary arms races that occur between primary pathogens and host immune responses.

Beyond its anti-bacterial role, itaconate exhibits immunomodulatory properties via several mechanisms, for an in-depth analysis this has been nicely reviewed elsewhere^150,176,177^. Initially recognised as an anti-inflammatory metabolite for its ability to inhibit complex II^145^, itaconate has since been identified as a mildly electrophilic compound capable of alkylating protein cysteine thiols^164^, a process termed 2,3-dicarboxypropylation, and glutathione^178^. In addition, itaconate has also been identified as a competitive inhibitor of the TET family of α-KG-dependent DNA dioxygenases^179^ and a ligand of the α-KG receptor OXGR1^180^. The mild electrophilic nature of itaconate enables derivatives, such as dimethyl itaconate (DMI) or 4-octyl itaconate (4-OI), to modify various metabolic enzymes, redox regulators, and immune proteins^150,176^. Target modification by itaconate derivatives leads to activation of the anti-oxidant and stress-responsive transcription factors nuclear factor erythroid 2-related factor 2 (NRF2) and activating transcription factor 3 (ATF3), which in turn can limit pro-inflammatory cytokines such as IL-1β and IL-6^164,178^. NRF2 stabilisation is also decreased in IRG1-deficient macrophages and Kupffer cells under certain contexts suggesting a role for endogenous itaconate in NRF2 stabilisation^178,181,182^. However, treatment with underivatised itaconate has mixed results with regard to NRF2, increasing stability in some instances^183^, but not in others^43^ for unclear reasons. Furthermore, itaconate derivatives also alkylate key enzymes of glycolysis, including fructose-bisphosphate aldolase A (ALDOA) and glyceraldehyde 3-phosphate dehydrogenase (GAPDH), thereby curbing aerobic glycolysis associated with pro-inflammatory macrophage activation^184,185^. The list of targets modified by itaconate derivatives continues to grow and current data indicates they may represent a novel class of anti-inflammatory agents with clinical potential during infection and inflammatory disease^176,177^. Itaconate accumulation, in combination with NO, is also an important mediator of innate immune tolerance limiting NLRP3 inflammasome activation and pyroptosis through mechanisms dependent on complex II inhibition or it’s electrophilic properties^186–188^. Furthermore, myeloid-derived IRG1 dampens neutrophil-mediated lung inflammation following *M. tuberculosis* infection, underscoring the importance of itaconate in vivo^189^. However, following trauma, itaconate-producing neutrophils play an important role in tissue inflammation and the wound healing process following tendon injury^190^. In contrast, itaconate production in DCs impairs anti-parasitic immune responses by promoting mtDNA-dependent PD-L1 expression following *Plasmodium chabaudi* infection, which limits CD8^+^ T cells^191^. This suggests that the beneficial effects of itaconate synthesis may vary depending on the pathogen involved. In summary, these studies highlight the importance of the mitochondrial IRG1-itaconate axis in regulating the innate immune response to pathogens, and for the most part, in restricting hyperinflammatory responses. However, the relative importance of endogenous itaconate cysteine reactivity versus metabolic perturbations during an immune response remains to be determined and requires further investigation.

Similarly to itaconate, fumarate is a mildly electrophilic metabolite that can modify protein cysteine thiols and glutathione, a process termed succination^138^. Fumarate levels increase following inflammatory macrophage activation in a mechanism dependent on glutamine anaplerosis and induction of aspartate-arginosuccinate shunt^138,144^. Inhibiting this shunt limits arginine synthesis and leads to a reduction in pro-inflammatory mediators, including NO and IL-6^144^. Fumarate accumulation can also enhance TNF-α production by inhibiting autocrine IL-10 signalling in macrophages^138^ and inhibiting lysine demethylase 5 (KDM5) histone demethylases in monocytes^192^. Furthermore, fumarate accumulation has also been implicated in anti-bacterial defence owing to its cysteine reactivity^35^, which can intoxicate pathogens including *Mycobacterium tuberculosis*^193^. The intricate interactions between the TCA cycle and innate immunity underscore the significance of metabolic reprogramming in shaping immune responses. Understanding the regulatory roles of TCA cycle intermediates, such as succinate and itaconate, opens new avenues for therapeutic interventions using immunomodulatory metabolite derivatives.

### Cardiolipin signalling

Cardiolipin is a unique phospholipid found predominantly in the IMM of eukaryotic cells and can be found in most bacterial species^194^. Structurally, it consists of two phosphatidyl groups linked by a glycerol backbone, resulting in a dimeric structure. The presence of four acyl chains contributes to its distinctive conical shape, which promotes curvature of the membrane and cristae morphology^194^. Approximately 10-15% of all mitochondrial phospholipid content is cardiolipin^194^. Cardiolipin stabilises the respiratory chain complexes to support mitochondrial bioenergetics, whilst also being implicated in protein import, mitophagy, apoptosis and mitochondrial dynamics^194^. In addition to these identified functions, cardiolipin is emerging as a regulator of innate immune signalling and inflammatory cell death.

Cardiolipin found in human serum has been observed to have an interesting role in immune regulation^195^. It has been reported to promote the surface expression of the non-polymorphic major histocompatibility complex (MHC) class I-like molecule CD1d in DCs, a process that relies on peroxisome proliferator-activated receptor (PPAR) nuclear hormone receptors^195^. Furthermore, CD1d is capable of binding to bacterial and eukaryotic cardiolipin and when presented by DCs, can activate splenic and hepatic γδ T cells in vivo^196^. These findings suggest that DCs play a crucial role in antigen presentation of bacterial cardiolipin following infection or mitochondrial cardiolipin following tissue injury, which may represent a key immunosurveillance mechanism.

**Fig. 4:**
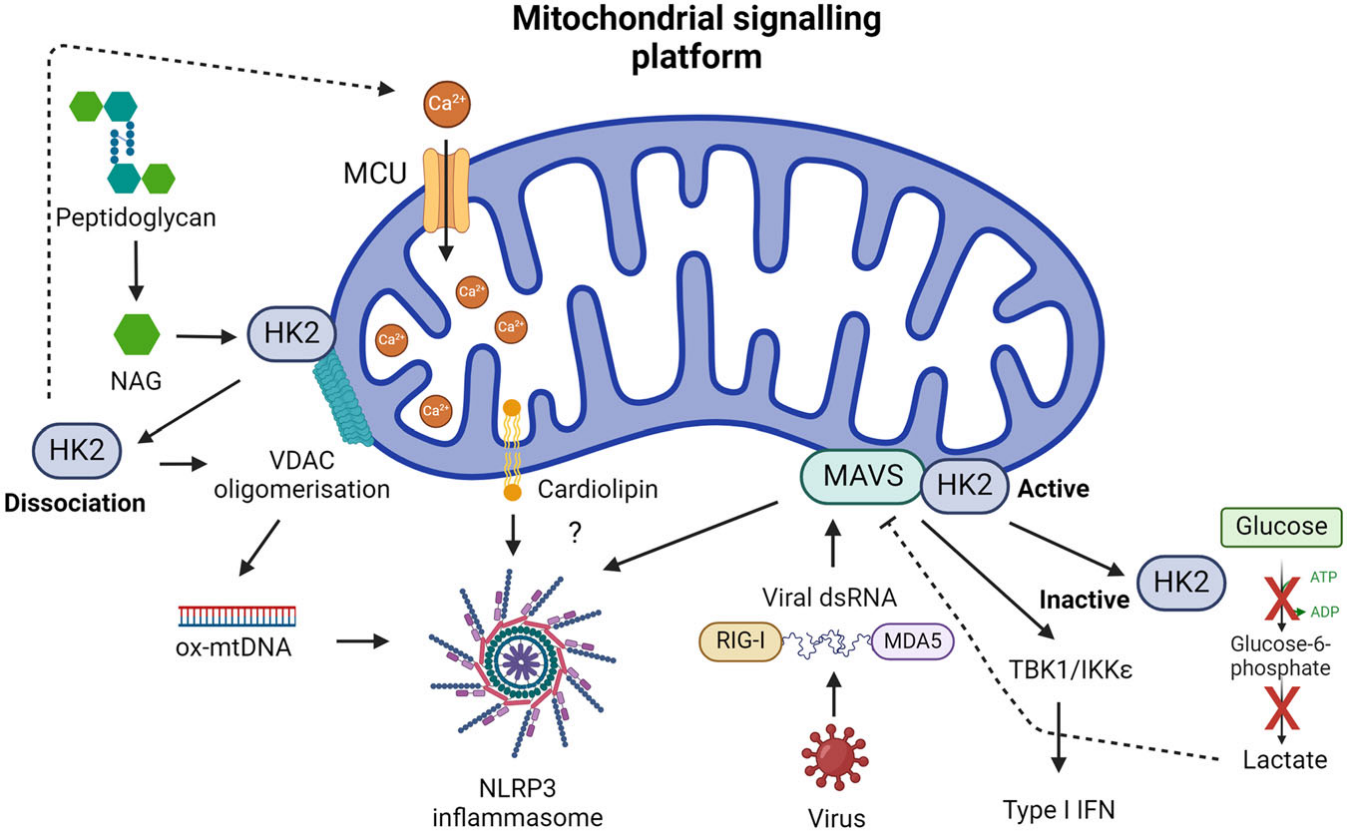
Mitochondrial signalling platform in innate immunity. MAVS localized to the OMM integrates the sensing of viral RNA by RIG-I or MDA5 to trigger a type I IFN response and recruit NLRP3 to mitochondria, an event also attributed to cardiolipin. HK2 association with MAVS and mitochondria promotes its enzymatic activity, which can lead to increased lactate and MAVS inhibition. An inhibition that’s relieved when HK2 dissociates from mitochondria. HK2 can also associate with VDAC and sense NAG, a breakdown product of bacterial peptidoglycan. HK2 dissociation from VDAC promotes mitochondrial Ca^2+^ uptake, VDAC oligomerisation, and mtDNA release to activate the NLRP3 inflammasome.

In macrophages, cardiolipin has been implicated in supporting NLRP3 inflammasome activation (Fig. 4)^197^. Research suggests that cardiolipin interacts with NLRP3 after translocation to OMM, indicating that the outer membrane is a critical site for co-ordinating NLRP3 signalling^197^. Notably, NLRP3 activation was hindered when cardiolipin synthase (CSL) was genetically silenced^197^. Recent findings also indicate that GSDMD causes mitochondrial damage by permeabilising both the IMM and OMM^69,198,199^. Mechanistically, impairing cardiolipin biosynthesis or the transfer of cardiolipin to the OMM by the scramblase PLSCR3 prevented GSDMD recruitment and subsequent pyroptosis^198^. However, high-resolution structures of NLRP3-activated ASC complexes using cryo-electron tomography do not show co-localisation with mitochondria, despite supporting GSDMD-mediated mitochondrial pore formation^199^. This data suggests that NLRP3 signalling at the OMM may not occur as previously suggested. However, it’s important to note that an earlier interaction between NLRP3 and the OMM, which may not have been captured in the structure, cannot be conclusively ruled out.

Furthermore, Reynolds et al. (2023) reported that loss of cardiolipin biosynthesis, achieved by silencing CSL in macrophages, also impaired *Il1b* expression via a complex II-dependent mechanism^26^. This indicates that a loss of cardiolipin could more broadly impact the pro-inflammatory response and limit NLRP3-mediated IL-β release by reducing pro-IL-1β levels. Indeed, the importance of cardiolipin in mitochondrial respiration and cristae architecture presents a challenge in distinguishing its role in bioenergetics from its involvement in OMM signalling when its biosynthesis is disrupted. Mitochondrial respiration relies on the proper functioning of respiratory chain complexes embedded within the IMM, where cardiolipin plays a crucial role in stabilising and optimising their activity. Disruption of cardiolipin biosynthesis can impair mitochondrial respiration, affecting cellular ATP production and potentially influencing NLRP3 inflammasome activation, as bioenergetics status is a known regulator of this process^38^. As such, the precise role of cardiolipin is unclear and will require sophisticated experimental approaches to disentangle.

## Mitochondria as a signalling platform

A key facet of mitochondrial signal transduction is found in the organelles ability to function as a scaffold, thereby facilitating cellular signalling cascades. Central to this paradigm is mitochondrial antiviral signalling protein (MAVS), also known as IFNβ promoter stimulator 1 (IPS1), CARD adaptor inducing IFNβ (CARDIF) and virus-induced signalling adaptor (VISA), a key mediator that interfaces with RIG-I-like receptors (RLRs)^200^. As such, MAVS serves as a critical nexus for the integration of intracellular antiviral signalling (Fig. 4)^201^. Structurally, MAVS is a 540-amino acid protein comprising three distinct functional domains: an N-terminal CARD domain, a proline-rich region, and a C-terminal transmembrane domain. The N-terminal CARD domain of MAVS facilitates interaction with the CARD domains of RLRs, including RNA helicases RIG-I and MDA5^202^. This interaction is pivotal for initiating signalling events leading to the release of type I IFN and the activation of the NF-κB and IRF pathways^203–205^. The subcellular localisation of MAVS adds an additional layer of complexity to its function. MAVS dynamically associates with the OMM, endoplasmic reticulum, and peroxisomes, suggesting a versatile role in coordinating antiviral responses across distinct cellular compartments^206^. Upon viral infection, peroxisomal MAVS induces the rapid IFN-independent expression of defence factors that provide short-term protection, whereas mitochondrial MAVS activates a delayed IFN-dependent signalling pathway, which amplifies and stabilises the antiviral response^206^.

Upon activation, MAVS undergoes oligomerisation, thereby forming fibrils that induce membrane remodelling and signalling complex assembly^207,208^. Independent of RLR sensing of RNA, mtROS can promote MAVS oligomerisation and type I IFN production in SLE patients^207^, which suggest MAVS may act as a mitochondrial redox sensor. Like cardiolipin, MAVS is also reported to recruit the NLRP3 inflammasome to mitochondria, thereby triggering its activation^209,210^. This appears to occur when using standard NLRP3 stimuli^209^ and in response to Sendai Virus, also known as murine respirovirus, infection^210^. Notably, while a structural study failed to capture an interaction between NLRP3 and mitochondria using cryo-electron ET^199^, this study was not conducted in the context of viral infection. Therefore, it remains possible that NLRP3 interacts with mitochondria following viral sensing and MAVS oligomerisation, an aspect that warrants further investigation.

The localisation of MAVS to the mitochondrial membrane suggests a potential interplay with mitochondrial dynamics within macrophages. Recent studies propose that MAVS may influence mitochondrial morphology and function, thereby modulating the metabolic profile of macrophages during the course of an antiviral response. Specifically, the fusion mechanisms of the OMM are rigorously governed by Mitofusin 1 (Mfn1) and Mitofusin 2 (Mfn2), exerting regulatory control over MAVS activity^211^. Surprisingly, while Mfn1 positively regulates MAVS-mediated antiviral responses, its close homolog Mfn2 directly inhibits MAVS, possibly unrelated to its function in mitochondrial dynamics^212,213^. Thus, although Mfn1 and Mfn2 share the function of inducing mitochondrial fusion, they play opposing roles in viral innate immunity. Mitochondrial dynamics, especially mitochondrial fusion, appears crucial for the innate immune response. Conversely, promoting mitochondrial fission, via dynamin-related protein 1 (DRP1), inhibits MAVS activity during viral infection^214,215^.

Hexokinase 2 (HK2), a key enzyme in glucose metabolism has recently been identified as a novel interactor with MAVS (Fig. 4)^216,217^. When MAVS is inactive, it forms a complex with HK2, inducing its localisation to the mitochondria, where it associates with the OMM through its interaction with the voltage-dependent anion channel (VDAC), and maintaining its enzymatic activity^216^. RLR signalling disrupts glucose metabolism, leading to the downregulation of glycolysis. Mechanistically, MAVS, in its active state, binds to RIG-I, releasing HK2 into the cytoplasm, impairing its activity and subsequent glucose metabolism. HK2 inactivation leads to the decrease of intracellular lactate levels, which can inhibit RLR/MAVS signalling^216^. This intricate regulation suggests a role for the MAVS-HK2 axis in connecting the innate immune response with cellular bioenergetics during viral challenges. In macrophages, HK2 is also reported to associate with VDAC on mitochondria to act as an innate immune sensor for bacterial peptidoglycan^218^. Phagosomal processing of peptidoglycan leads to the release of N-acetylglucosamine (NAG) that inhibits HK2 triggering its dissociation from the OMM and activates NLRP3^218^. Mechanistically, HK2 dissociation from the OMM promotes mitochondrial Ca^2+^ uptake, VDAC oligomerisation and the release of mtDNA^219^. Finally, in DCs, TLR activation promotes HK2 association with mitochondria to facilitate the rapid induction of glycolysis, which was essential for DC activation^32^.

In summary, the mitochondrial signalling platform, often centred around MAVS, HK2 and VDAC, serve as a crucial nexus orchestrating innate immune responses against bacterial and viral infections. The convergence of mitochondrial dynamics and antiviral signalling pathways underscores the intricate cellular mechanisms deployed to counteract pathogenic threats. Future research endeavours focused on unravelling the complexities of RLR recruitment to mitochondria are poised to enhance our comprehension of this vital axis in innate immunity. Collectively, the studies on NLRP3 also highlight how all facets of mitochondrial physiology are intertwined and work together to drive activation of this complicated signalling complex.

## Future outlook and concluding remarks

Much of the research conducted thus far has involved extensive in vitro stimulations of bone marrow- or monocyte-derived macrophages, DCs, and neutrophils to model in vivo cell populations. While these model systems are valuable for studying innate immune cell biology, they do not precisely replicate tissue-resident or infiltrating in vivo cell populations. The latter are often shaped by a complex and dynamic microenvironment that is difficult to reproduce in vitro^220^. However, there are now expanding toolkits emerging that will facilitate the measurement of metabolic genes and metabolism in immune cells in vitro and in vivo. Experimental changes to the medium composition and cell culture geometry can now more closely reproduce in vivo conditions without over complicating experimental methodologies^221–223^. Significant progress has been made in single-cell techniques, including single-cell RNA sequencing (scRNA-seq), which has been used to identify OxPhos as a distinguishing feature of tissue-resident macrophages across different organs under steady state and obesogenic conditions^224^. High-dimensional spectral flow cytometry has also identified tissue-resident macrophage metabolic heterogeneity during helminth infection^225^. Other emerging techniques, such as single-cell energetic metabolism by profiling translation inhibition (SCENITH), allow the study of energy metabolism using flow cytometry and have been applied to in vitro and ex vivo human and murine myeloid populations^226,227^. Finally, progress is also being made in mass spectrometry imaging (MSI), which has been applied for joint protein-metabolite profiling of single immune and cancer cells^228^. These expanding metabolic toolkits will enable greater investigations of mitochondrial metabolism and signalling in innate immune cell populations and beyond.

Since the designation of mitochondria as the ‘powerhouse of the cell’, further research has revealed, as discussed here, their role as centrally positioned signalling hubs essential for innate immune signalling. However, while the importance of mitochondria cannot be overstated, many aspects of how they influence innate immune function remain unclear. There remain many outstanding questions to be addressed in future work to better understand the role of mitochondria in innate immunity. This is exemplified by the NLRP3 inflammasome, which is evidently regulated by mitochondrial function (Table 1). What is the precise role of specific mitochondrial signals such as mtROS, cardiolipin, and ATP synthesis for the activation of this inflammatory signalling complex during bacterial and viral infection? And are these involved in NLRP3 activation in vivo? What is the source of mtROS and is it dependent on RET?^56^ The answer to these outstanding questions may aid with therapeutic targeting of this process during infection or inflammatory disease. Finally, given the importance of mitochondria to innate immune cell biology, to what extent are mitochondrial diseases a manifestation of innate immune cell dysfunction?^229^ Or what proportion of more common autoimmune disorders are driven by a break in mitochondrial endosymbiosis?^7^ We hope this review will inspire research into these and many other questions that remain to be explored and will promote a clearer comprehension of the extensive role of mitochondria in innate immunity.

**Table 1:** Mitochondrial signal transduction and NLRP3 activation.

Mitochondrial signal	Signalling	Outcome	Cell type
ATP and PCr synthesis	Cytosolic ATP synthesis by CKB	NLRP3 activation, IL-1β	Macrophage
mtROS (Complex I-derived)	HIF-1α stabilisation and *Il1b* transcription	NLRP3 activation, IL-1β	Macrophage
mtDNA, mtROS	Release of ox-mtDNA fragments	NLRP3 activation, IL-1β	Macrophage
Itaconate	Complex II inhibition, electrophilic properties	Inhibition of NLRP3 and pyroptosis	Macrophage
Cardiolipin	Biosynthesis and translocation to OMM	NLRP3 recruitment and activation, IL-1β, pyroptosis	Macrophage
MAVS	Oligomerisation on OMM	NLRP3 recruitment and activation, IL-1β	Macrophage
Mfn1	MAVS activation	NLRP3 activation, IL-1β	Macrophage
Mfn2	MAVS inhibition	NLRP3 inhibition	Macrophage
DRP1	MAVS inhibition	NLRP3 inhibition	Macrophage
HK2 dissociation from OMM	Mitochondrial Ca^2+^ uptake increase, VDAC oligomerisation	mtDNA release, NLRP3 activation, IL-1β	Macrophage
